# 2,4-Bis(2-fluoro­phen­yl)-3-aza­bicyclo­[3.3.1]nonan-9-one

**DOI:** 10.1107/S1600536809022065

**Published:** 2009-06-17

**Authors:** P. Parthiban, V. Ramkumar, Yeon Tae Jeong

**Affiliations:** a Division of Image Science and Information Engineering, Pukyong National University, Busan 608 739, Republic of Korea; b Department of Chemistry, IIT Madras, Chennai, TamilNadu, India

## Abstract

The title compound, C_20_H_19_F_2_NO, exists in a twin-chair conformation with an equatorial orientation of the two 2-fluoro­phenyl groups on both sides of the secondary amine group. The benzene rings are orientated at an angle of 25.68 (4)° with respect to one another and the F atoms point upwards (towards the carbonyl group). The crystal is stabilized by an inter­molecular N—H⋯π inter­action.

## Related literature

3-Aza­bicyclo­nona­nes are present in numerous naturally occurring diterpenoid/norditerpenoid alkaloids and display broad-spectrum biological activity, see: Hardick *et al.* (1996 ▶); Jeyaraman *et al.* (1981 ▶); For related structures, see: Parthiban *et al.* (2008*a* ▶,*b* ▶, 2009 ▶); Parthiban, Ramkumar, Kim *et al.* (2008 ▶); Parthiban, Ramkumar, Santan *et al.* (2008 ▶); Parthiban, Thirumurugan *et al.* (2008 ▶). For puckering parameters, see: Cremer & Pople (1975 ▶).
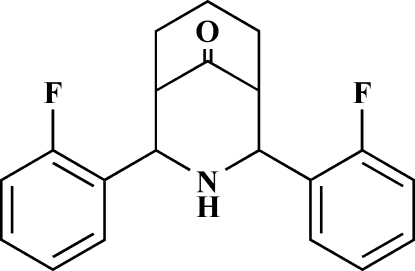


## Experimental

### 

#### Crystal data

C_20_H_19_F_2_NO
*M* *_r_* = 327.36
Triclinic, 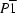

*a* = 7.4699 (3) Å
*b* = 10.6621 (4) Å
*c* = 10.7131 (4) Å
α = 78.027 (2)°
β = 78.946 (2)°
γ = 87.201 (2)°
*V* = 819.16 (5) Å^3^
*Z* = 2
Mo *K*α radiation
μ = 0.10 mm^−1^
*T* = 298 K
0.42 × 0.38 × 0.12 mm

#### Data collection

Bruker APEXII CCD area-detector diffractometer
Absorption correction: multi-scan (*SADABS*; Bruker, 1999 ▶) *T*_min_ = 0.960, *T*_max_ = 0.989
11219 measured reflections
3913 independent reflections
2564 reflections with *I* > 2σ(*I*)
*R*_int_ = 0.021

#### Refinement

*R*[*F* ^2^ > 2σ(*F* ^2^)] = 0.045
*wR*(*F* ^2^) = 0.156
*S* = 0.81
3913 reflections
221 parameters
H atoms treated by a mixture of independent and constrained refinement
Δρ_max_ = 0.17 e Å^−3^
Δρ_min_ = −0.20 e Å^−3^

### 

Data collection: *APEX2* (Bruker, 2004 ▶); cell refinement: *SAINT-Plus* (Bruker, 2004 ▶); data reduction: *SAINT-Plus*; program(s) used to solve structure: *SHELXS97* (Sheldrick, 2008 ▶); program(s) used to refine structure: *SHELXL97* (Sheldrick, 2008 ▶); molecular graphics: *ORTEP-3* (Farrugia, 1997 ▶); software used to prepare material for publication: *SHELXL97*.

## Supplementary Material

Crystal structure: contains datablocks global, I. DOI: 10.1107/S1600536809022065/bq2146sup1.cif

Structure factors: contains datablocks I. DOI: 10.1107/S1600536809022065/bq2146Isup2.hkl

Additional supplementary materials:  crystallographic information; 3D view; checkCIF report

## Figures and Tables

**Table 1:** Hydrogen-bond geometry (Å, °)

*D*—H⋯*A*	*D*—H	H⋯*A*	*D*⋯*A*	*D*—H⋯*A*
N1—H1*A*⋯*Cg*^i^	0.90 (4)	2.72 (2)	3.58 (16)	167.3 (19)

Symmetry code: (i) 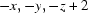. *Cg* is the centroid of C9–C14 phenyl ring.

**Fig. 1.**
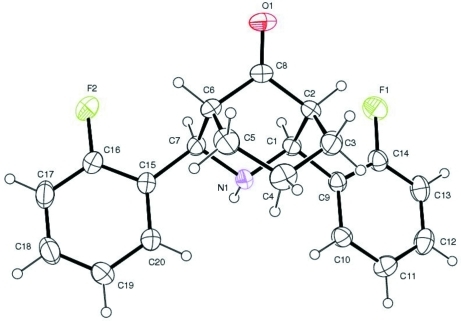
The molecular structure of title compound, showing 30% probability displacement ellipsoids.

**Fig. 2.**
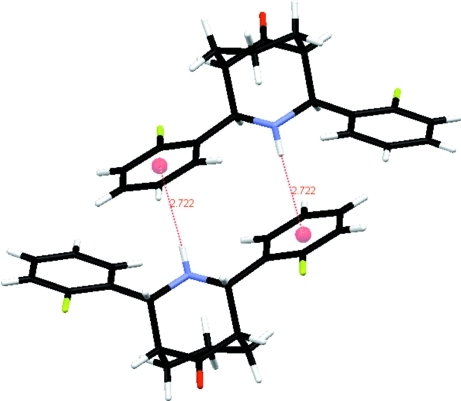
The packing diagram of title compound showing N—H···π interaction

## supplementary crystallographic information

### Comment

3-Azabicyclononanes are important class of heterocycles due to their presence in numerous naturally occurring diterpenoid/norditerpenoid alkaloids and broad spectrum biological activities (Jeyaraman & Avila, 1981; Hardick *et al.*, 1996). Since the stereochemistry plays crucial role in exploiting biological activities, it is essential to establish the stereochemistry of the bio-active molecules. Irrespective of the nature and position of the substituents on the phenyl, similar compounds show twin-chair conformation (Parthiban *et al.* (2008*a*,*b*, 2009; Parthiban, Ramkumar, Kim *et al.* (2008), Parthiban, Ramkumar, Santan*et al.*, 2008; ; Parthiban, Thirumurugan *et al.*,2008). However, to explore the impact of fluorine atom, substituted at *ortho* position of the phenyl groups on both sides of the hetero atom, we have carried out the single-crystal x-ray diffraction study for the title compound.

The title compound C_20_H_19_F_2_NO, (I), exists in twin-chair conformation with equatorial orientation of the *ortho*-fluorophenyl group on both sides of the secondary amino group with the torsion angle of C8—C2—C1—C9 and C8—C6—C7—C15 as 179.99 (3) and 179.48 (4)°, respectively. The aryl groups are orientated at an angle of 25.68 (4)° to each other. In both aryl groups, the F atom is pointed towards the carbonyl group (Figure 1.). Analysis of torsion angles, asymmetry parameters and least-squares plane calculation shows that the piperidine ring adopts near ideal chair conformation with the deviation of ring atoms N1 and C8 from the C1/C2/C6/C7 plane by -0.654 (3) Å and 0.696 (3) Å, respectively; Q_T_ = 0.6002 (18) Å, q(2)= 0.0242 (17) Å, q(3)=-0.5996 (18) Å, θ = 177.54 (16)° whereas the cyclohexane ring atoms C4 and C8 deviate from the C2/C3/C5/C6 plane by -0.529 (4) Å and 0.727 (3) Å, respectively; Q_T_ = 0.565 (2) Å, q(2)= 0.146 (2) Å, q(3)= -0.546 (2) Å, θ = 165.1 (2)° (Cremer & Pople, 1975). Hence, the title compound (I) shows appreciable deviation from the ideal chair conformation of the cyclohexane moiety. The crystal structure is stabilized by intermolecular N—H···π interaction (Figure 2.).

### Experimental

A mixture of cyclohexanone (0.025 mol, 2.45 g) and *ortho*-fluorobenzaldehyde (0.05 mol, 6.21 g) was added to a warm solution of ammonium acetate (0.04 mol, 3.08 g) in 30 ml of absolute ethanol. The mixture was gently warmed with stirring till the yellow color was formed during the mixing of the reactants and then stirred at room temperature up to the formation of product. At the end, the crude azabicyclic ketone was separated by filtration and washed with 1:5 ethanol-ether mixture to remove the coloring impurities. Recrystallization of the compound from acetone gave X-ray diffraction quality crystals of 2,4-bis(2-fluorophenyl)-3-azabicyclo[3.3.1]nonan-9-one.

### Refinement

Nitrogen H atoms were located in a difference Fourier map and refined isotropically. Other hydrogen atoms were fixed geometrically and allowed to ride on the parent carbon atoms, with aromatic C—H =0.93 Å, aliphatic C—H = 0.98 Å and methylen C—H = 0.97 Å. The displacement parameters were set for phenyl, methylen and aliphatic H atoms at *U*_iso_(H) = 1.2*U*_eq_(C).

### Crystal data

**Table:**

C_20_H_19_F_2_NO	*Z* = 2
*M**_r_* = 327.36	*F*(000) = 344
Triclinic, *P*1	*D*_x_ = 1.327 Mg m^−^^3^
Hall symbol: -P 1	Mo *K*α radiation, λ = 0.71073 Å
*a* = 7.4699 (3) Å	Cell parameters from 3420 reflections
*b* = 10.6621 (4) Å	θ = 2.5–27.4°
*c* = 10.7131 (4) Å	µ = 0.10 mm^−^^1^
α = 78.027 (2)°	*T* = 298 K
β = 78.946 (2)°	Block, colourless
γ = 87.201 (2)°	0.42 × 0.38 × 0.12 mm
*V* = 819.16 (5) Å^3^	

### Data collection

**Table:**

Bruker APEXII CCD area-detector diffractometer	3913 independent reflections
Radiation source: fine-focus sealed tube	2564 reflections with *I* > 2σ(*I*)
graphite	*R*_int_ = 0.021
φ and ω scans	θ_max_ = 28.5°, θ_min_ = 2.0°
Absorption correction: multi-scan (*SADABS*; Bruker, 1999)	*h* = −9→9
*T*_min_ = 0.960, *T*_max_ = 0.989	*k* = −14→14
11219 measured reflections	*l* = −14→14

### Refinement

**Table:**

Refinement on *F*^2^	Primary atom site location: structure-invariant direct methods
Least-squares matrix: full	Secondary atom site location: difference Fourier map
*R*[*F*^2^ > 2σ(*F*^2^)] = 0.045	Hydrogen site location: inferred from neighbouring sites
*wR*(*F*^2^) = 0.156	H atoms treated by a mixture of independent and constrained refinement
*S* = 0.81	*w* = 1/[σ^2^(*F*_o_^2^) + (0.1*P*)^2^ + 0.2685*P*] where *P* = (*F*_o_^2^ + 2*F*_c_^2^)/3
3913 reflections	(Δ/σ)_max_ < 0.001
221 parameters	Δρ_max_ = 0.17 e Å^−^^3^
0 restraints	Δρ_min_ = −0.20 e Å^−^^3^

### Special details

**Table:**

Geometry. All e.s.d.'s (except the e.s.d. in the dihedral angle between two l.s. planes)are estimated using the full covariance matrix. The cell e.s.d.'s are takeninto account individually in the estimation of e.s.d.'s in distances, anglesand torsion angles; correlations between e.s.d.'s in cell parameters are onlyused when they are defined by crystal symmetry. An approximate (isotropic)treatment of cell e.s.d.'s is used for estimating e.s.d.'s involving l.s. planes.
Refinement. Refinement of *F*^2^ against ALL reflections. The weighted *R*-factor *wR* and goodness of fit *S* are based on *F*^2^, conventional *R*-factors *R* are based on *F*, with *F* set to zero for negative *F*^2^. The threshold expression of *F*^2^ > σ(*F*^2^) is used only for calculating *R*-factors(gt) *etc*. and is not relevant to the choice of reflections for refinement. *R*-factors based on *F*^2^ are statistically about twice as large as those based on *F*, and *R*- factors based on ALL data will be even larger.

### Fractional atomic coordinates and isotropic or equivalent isotropic displacement parameters (Å^2^)

**Table:**

	*x*	*y*	*z*	*U*_iso_*/*U*_eq_	
C1	0.2684 (2)	0.16060 (16)	0.96799 (14)	0.0419 (4)	
H1	0.3499	0.0869	0.9585	0.050*	
C2	0.3813 (2)	0.27242 (18)	0.98432 (15)	0.0481 (4)	
H2	0.4408	0.2431	1.0594	0.058*	
C3	0.2715 (3)	0.39499 (18)	1.00204 (17)	0.0546 (5)	
H3A	0.1694	0.3730	1.0734	0.066*	
H3B	0.3485	0.4532	1.0261	0.066*	
C4	0.1990 (3)	0.46430 (18)	0.88289 (19)	0.0595 (5)	
H4A	0.1607	0.5502	0.8941	0.071*	
H4B	0.0925	0.4196	0.8758	0.071*	
C5	0.3381 (3)	0.47348 (18)	0.75758 (18)	0.0591 (5)	
H5A	0.4243	0.5403	0.7526	0.071*	
H5B	0.2755	0.4987	0.6847	0.071*	
C6	0.4445 (2)	0.34774 (19)	0.74473 (16)	0.0506 (4)	
H6	0.5433	0.3651	0.6689	0.061*	
C7	0.3290 (2)	0.23549 (16)	0.73240 (14)	0.0431 (4)	
H7	0.4101	0.1616	0.7234	0.052*	
C8	0.5261 (2)	0.3061 (2)	0.86420 (17)	0.0540 (5)	
C9	0.1194 (2)	0.12155 (15)	1.08599 (14)	0.0397 (4)	
C10	−0.0618 (2)	0.15942 (16)	1.08968 (16)	0.0463 (4)	
H10	−0.0971	0.2074	1.0153	0.056*	
C11	−0.1906 (3)	0.12692 (19)	1.20224 (18)	0.0560 (5)	
H11	−0.3111	0.1535	1.2026	0.067*	
C12	−0.1423 (3)	0.05585 (19)	1.31351 (17)	0.0595 (5)	
H12	−0.2294	0.0355	1.3891	0.071*	
C13	0.0352 (3)	0.01505 (18)	1.31262 (16)	0.0569 (5)	
H13	0.0693	−0.0347	1.3866	0.068*	
C14	0.1613 (2)	0.04921 (16)	1.20018 (15)	0.0470 (4)	
C15	0.2390 (2)	0.26888 (15)	0.61458 (14)	0.0415 (4)	
C16	0.3352 (2)	0.25928 (19)	0.49306 (16)	0.0524 (4)	
C17	0.2607 (3)	0.2853 (2)	0.38267 (16)	0.0638 (5)	
H17	0.3306	0.2767	0.3031	0.077*	
C18	0.0817 (3)	0.3240 (2)	0.39162 (17)	0.0641 (5)	
H18	0.0289	0.3419	0.3180	0.077*	
C19	−0.0185 (3)	0.3362 (2)	0.50969 (18)	0.0586 (5)	
H19	−0.1396	0.3630	0.5158	0.070*	
C20	0.0587 (2)	0.30888 (17)	0.62043 (15)	0.0483 (4)	
H20	−0.0116	0.3176	0.6998	0.058*	
F1	0.33764 (16)	0.01112 (12)	1.20093 (11)	0.0726 (4)	
F2	0.51212 (16)	0.21980 (16)	0.48284 (11)	0.0853 (4)	
N1	0.19010 (18)	0.19950 (13)	0.85064 (11)	0.0397 (3)	
O1	0.68765 (18)	0.30314 (19)	0.86537 (14)	0.0831 (5)	
H1A	0.129 (3)	0.1358 (19)	0.8399 (17)	0.050 (5)*	

### Atomic displacement parameters (Å^2^)

**Table:**

	*U*^11^	*U*^22^	*U*^33^	*U*^12^	*U*^13^	*U*^23^
C1	0.0392 (9)	0.0502 (9)	0.0351 (7)	0.0105 (7)	−0.0090 (6)	−0.0071 (6)
C2	0.0358 (9)	0.0718 (12)	0.0383 (8)	−0.0009 (8)	−0.0129 (7)	−0.0095 (7)
C3	0.0525 (11)	0.0631 (11)	0.0508 (9)	−0.0147 (8)	−0.0022 (8)	−0.0209 (8)
C4	0.0605 (12)	0.0489 (10)	0.0693 (12)	0.0008 (8)	−0.0086 (9)	−0.0159 (9)
C5	0.0642 (13)	0.0551 (11)	0.0563 (10)	−0.0146 (9)	−0.0138 (9)	−0.0020 (8)
C6	0.0322 (9)	0.0761 (12)	0.0394 (8)	−0.0063 (8)	−0.0011 (6)	−0.0058 (8)
C7	0.0383 (9)	0.0550 (9)	0.0346 (7)	0.0099 (7)	−0.0049 (6)	−0.0096 (6)
C8	0.0326 (9)	0.0790 (13)	0.0511 (9)	−0.0024 (8)	−0.0082 (7)	−0.0138 (9)
C9	0.0453 (9)	0.0394 (8)	0.0346 (7)	0.0037 (6)	−0.0101 (6)	−0.0068 (6)
C10	0.0446 (10)	0.0486 (9)	0.0438 (8)	0.0023 (7)	−0.0109 (7)	−0.0035 (7)
C11	0.0452 (10)	0.0608 (11)	0.0579 (10)	−0.0033 (8)	−0.0012 (8)	−0.0098 (8)
C12	0.0694 (14)	0.0598 (11)	0.0437 (9)	−0.0146 (10)	0.0050 (8)	−0.0087 (8)
C13	0.0797 (14)	0.0511 (10)	0.0377 (8)	−0.0041 (9)	−0.0130 (8)	−0.0011 (7)
C14	0.0542 (11)	0.0456 (9)	0.0423 (8)	0.0080 (7)	−0.0159 (7)	−0.0064 (7)
C15	0.0440 (9)	0.0463 (9)	0.0330 (7)	−0.0007 (7)	−0.0048 (6)	−0.0073 (6)
C16	0.0469 (10)	0.0682 (12)	0.0413 (8)	−0.0012 (8)	−0.0013 (7)	−0.0152 (8)
C17	0.0727 (14)	0.0846 (14)	0.0337 (8)	−0.0122 (11)	−0.0027 (8)	−0.0149 (8)
C18	0.0746 (14)	0.0775 (14)	0.0419 (9)	−0.0108 (11)	−0.0218 (9)	−0.0032 (9)
C19	0.0521 (11)	0.0728 (13)	0.0504 (10)	0.0027 (9)	−0.0182 (8)	−0.0036 (8)
C20	0.0469 (10)	0.0581 (10)	0.0377 (8)	0.0034 (8)	−0.0067 (7)	−0.0068 (7)
F1	0.0669 (8)	0.0863 (9)	0.0600 (7)	0.0256 (6)	−0.0257 (6)	0.0019 (6)
F2	0.0542 (7)	0.1445 (13)	0.0570 (7)	0.0195 (7)	0.0026 (5)	−0.0363 (7)
N1	0.0402 (8)	0.0478 (8)	0.0312 (6)	−0.0022 (6)	−0.0083 (5)	−0.0062 (5)
O1	0.0315 (8)	0.1455 (16)	0.0696 (9)	−0.0015 (8)	−0.0105 (6)	−0.0143 (9)

### Geometric parameters (Å, °)

**Table:**

C1—N1	1.4617 (19)	C9—C14	1.386 (2)
C1—C9	1.516 (2)	C9—C10	1.389 (2)
C1—C2	1.551 (3)	C10—C11	1.384 (2)
C1—H1	0.9800	C10—H10	0.9300
C2—C8	1.506 (2)	C11—C12	1.374 (3)
C2—C3	1.535 (3)	C11—H11	0.9300
C2—H2	0.9800	C12—C13	1.374 (3)
C3—C4	1.518 (3)	C12—H12	0.9300
C3—H3A	0.9700	C13—C14	1.374 (2)
C3—H3B	0.9700	C13—H13	0.9300
C4—C5	1.520 (3)	C14—F1	1.361 (2)
C4—H4A	0.9700	C15—C16	1.382 (2)
C4—H4B	0.9700	C15—C20	1.386 (2)
C5—C6	1.542 (3)	C16—F2	1.358 (2)
C5—H5A	0.9700	C16—C17	1.373 (3)
C5—H5B	0.9700	C17—C18	1.371 (3)
C6—C8	1.498 (2)	C17—H17	0.9300
C6—C7	1.550 (3)	C18—C19	1.368 (3)
C6—H6	0.9800	C18—H18	0.9300
C7—N1	1.4703 (19)	C19—C20	1.388 (2)
C7—C15	1.513 (2)	C19—H19	0.9300
C7—H7	0.9800	C20—H20	0.9300
C8—O1	1.208 (2)	N1—H1A	0.88 (2)
			
N1—C1—C9	110.56 (13)	O1—C8—C6	124.57 (16)
N1—C1—C2	109.45 (13)	O1—C8—C2	123.79 (16)
C9—C1—C2	110.71 (13)	C6—C8—C2	111.61 (14)
N1—C1—H1	108.7	C14—C9—C10	116.13 (14)
C9—C1—H1	108.7	C14—C9—C1	120.38 (14)
C2—C1—H1	108.7	C10—C9—C1	123.42 (13)
C8—C2—C3	107.64 (15)	C11—C10—C9	121.07 (15)
C8—C2—C1	107.86 (14)	C11—C10—H10	119.5
C3—C2—C1	114.94 (14)	C9—C10—H10	119.5
C8—C2—H2	108.8	C12—C11—C10	120.71 (18)
C3—C2—H2	108.8	C12—C11—H11	119.6
C1—C2—H2	108.8	C10—C11—H11	119.6
C4—C3—C2	114.55 (14)	C13—C12—C11	119.71 (16)
C4—C3—H3A	108.6	C13—C12—H12	120.1
C2—C3—H3A	108.6	C11—C12—H12	120.1
C4—C3—H3B	108.6	C14—C13—C12	118.66 (16)
C2—C3—H3B	108.6	C14—C13—H13	120.7
H3A—C3—H3B	107.6	C12—C13—H13	120.7
C3—C4—C5	113.26 (16)	F1—C14—C13	118.33 (15)
C3—C4—H4A	108.9	F1—C14—C9	117.96 (15)
C5—C4—H4A	108.9	C13—C14—C9	123.70 (16)
C3—C4—H4B	108.9	C16—C15—C20	116.11 (15)
C5—C4—H4B	108.9	C16—C15—C7	120.57 (15)
H4A—C4—H4B	107.7	C20—C15—C7	123.31 (13)
C4—C5—C6	114.00 (15)	F2—C16—C17	118.29 (15)
C4—C5—H5A	108.8	F2—C16—C15	118.14 (15)
C6—C5—H5A	108.8	C17—C16—C15	123.57 (17)
C4—C5—H5B	108.8	C18—C17—C16	118.98 (16)
C6—C5—H5B	108.8	C18—C17—H17	120.5
H5A—C5—H5B	107.6	C16—C17—H17	120.5
C8—C6—C5	107.17 (15)	C19—C18—C17	119.53 (17)
C8—C6—C7	107.89 (15)	C19—C18—H18	120.2
C5—C6—C7	115.27 (14)	C17—C18—H18	120.2
C8—C6—H6	108.8	C18—C19—C20	120.73 (18)
C5—C6—H6	108.8	C18—C19—H19	119.6
C7—C6—H6	108.8	C20—C19—H19	119.6
N1—C7—C15	110.02 (13)	C15—C20—C19	121.07 (15)
N1—C7—C6	109.92 (13)	C15—C20—H20	119.5
C15—C7—C6	111.89 (13)	C19—C20—H20	119.5
N1—C7—H7	108.3	C1—N1—C7	112.94 (12)
C15—C7—H7	108.3	C1—N1—H1A	109.7 (12)
C6—C7—H7	108.3	C7—N1—H1A	106.9 (12)
			
N1—C1—C2—C8	−57.90 (16)	C9—C10—C11—C12	−0.1 (3)
C9—C1—C2—C8	179.98 (13)	C10—C11—C12—C13	−0.9 (3)
N1—C1—C2—C3	62.16 (17)	C11—C12—C13—C14	1.4 (3)
C9—C1—C2—C3	−59.96 (17)	C12—C13—C14—F1	178.41 (16)
C8—C2—C3—C4	52.2 (2)	C12—C13—C14—C9	−0.9 (3)
C1—C2—C3—C4	−68.03 (19)	C10—C9—C14—F1	−179.44 (15)
C2—C3—C4—C5	−43.7 (2)	C1—C9—C14—F1	−2.3 (2)
C3—C4—C5—C6	44.4 (2)	C10—C9—C14—C13	−0.1 (3)
C4—C5—C6—C8	−53.8 (2)	C1—C9—C14—C13	177.01 (16)
C4—C5—C6—C7	66.3 (2)	N1—C7—C15—C16	−155.80 (16)
C8—C6—C7—N1	56.93 (17)	C6—C7—C15—C16	81.71 (19)
C5—C6—C7—N1	−62.77 (17)	N1—C7—C15—C20	23.5 (2)
C8—C6—C7—C15	179.48 (13)	C6—C7—C15—C20	−98.96 (18)
C5—C6—C7—C15	59.78 (17)	C20—C15—C16—F2	−179.68 (16)
C5—C6—C8—O1	−113.1 (2)	C7—C15—C16—F2	−0.3 (3)
C7—C6—C8—O1	122.2 (2)	C20—C15—C16—C17	−0.9 (3)
C5—C6—C8—C2	64.94 (19)	C7—C15—C16—C17	178.47 (18)
C7—C6—C8—C2	−59.75 (19)	F2—C16—C17—C18	179.38 (18)
C3—C2—C8—O1	113.9 (2)	C15—C16—C17—C18	0.6 (3)
C1—C2—C8—O1	−121.6 (2)	C16—C17—C18—C19	0.1 (3)
C3—C2—C8—C6	−64.20 (19)	C17—C18—C19—C20	−0.4 (3)
C1—C2—C8—C6	60.36 (19)	C16—C15—C20—C19	0.5 (3)
N1—C1—C9—C14	162.27 (15)	C7—C15—C20—C19	−178.81 (17)
C2—C1—C9—C14	−76.27 (19)	C18—C19—C20—C15	0.1 (3)
N1—C1—C9—C10	−20.8 (2)	C9—C1—N1—C7	−178.60 (13)
C2—C1—C9—C10	100.64 (18)	C2—C1—N1—C7	59.20 (17)
C14—C9—C10—C11	0.6 (2)	C15—C7—N1—C1	177.51 (13)
C1—C9—C10—C11	−176.38 (16)	C6—C7—N1—C1	−58.84 (17)

### Hydrogen-bond geometry (Å, °)

**Table:**

*D*—H···*A*	*D*—H	H···*A*	*D*···*A*	*D*—H···*A*
N1—H1A···Cg^i^	0.90 (4)	2.72 (2)	3.58 (16)	167.3 (19)

Symmetry codes: (i) −*x*, −*y*, −*z*+2.
